# Infective endocarditis in the real world: Diagnostic challenges and predictors of in-hospital mortality in a 10-year retrospective cohort from a Brazilian tertiary center

**DOI:** 10.1590/S1678-9946202668037

**Published:** 2026-06-12

**Authors:** Raquel Couto e Silva, Danilo Bortolotto Gurian, Guilherme Barbosa Pinto, Murilo Freua Sequeira, Erika Yukie Ishigaki, Eloisa Basile Siqueira Ayub, Nicoly Caroline de Andrade Delmondes, Gabriel Fialkovitz, Rinaldo Focaccia Siciliano, Olavo Henrique Munhoz Leite, Marcello Mihailenko Chaves Magri

**Affiliations:** 1 Centro Universitário FMABC, Disciplina de Infectologia, Santo André, São Paulo, Brazil; 2 Centro Universitário FMABC, Disciplina de Cardiologia, Santo André, São Paulo, Brazil; 3 Universidade de São Paulo, Faculdade de Medicina, Hospital das Clínicas, Instituto do Coração, Departamento de Infecção Hospitalar, São Paulo, São Paulo, Brazil; 4 Universidade de São Paulo, Faculdade de Medicina, Hospital das Clínicas, Divisão de Clínica de Moléstias Infecciosas e Parasitárias, São Paulo, São Paulo, Brazil

**Keywords:** Infective endocarditis, Staphylococcus aureus, Echocardiography, Blood cultures, Cardiac surgery, Mortality

## Abstract

Infective endocarditis (IE) remains a life-threatening condition with high mortality, particularly in middle-income countries where access to advanced diagnostics and timely surgery is unequal. Real-world data from such settings remain limited. This study describes the clinical and microbiological characteristics of patients with suspected IE and identifies independent predictors of in-hospital mortality at a Brazilian tertiary referral center. We conducted a retrospective cohort including adults hospitalized with suspected IE between 2012 and 2022. Cases were classified according to modified Duke criteria. Demographic, clinical, laboratory, microbiological, and echocardiographic data were collected, and independent predictors of in-hospital mortality were identified using multivariable logistic regression. Of the 112 patients evaluated, 66 met the criteria for definite or possible IE. Median age was 55 years, 59.1% were male, and 34.8% were ≥60 years. Structural heart disease was present in 87.8%, predominantly involving the mitral valve. Blood cultures were positive in 57.1%, with Staphylococcus aureus as the leading pathogen (25%), whereas 42.9% were culture-negative. Echocardiographic evidence of IE was documented in 80.9%, and valve surgery was performed in 51.5%. In-hospital mortality was 36.6%. Independent predictors of death included age ≥60 years (OR 7.0), hypertension (OR 3.9), new valvular regurgitation (OR 6.3), thrombocytopenia ≤150×10³/mm³ (OR 3.5), total bilirubin ≥1.2 mg/dL (OR 15.4), and arterial lactate >1.6 mmol/L (OR 5.2). In this real-world cohort, IE was characterized by diagnostic limitations, a high proportion of culture-negative cases, and high in-hospital mortality. Readily available clinical and laboratory parameters showed prognostic value and may support early risk stratification in resource-limited settings.

## INTRODUCTION

Infective endocarditis (IE) is a life-threatening infection that affects the endocardial surface of the heart, typically involving cardiac valves. Despite advances in antimicrobial therapy and diagnostic imaging, IE continues to pose major clinical challenges, with in-hospital mortality ranging from 15% to 30% globally^1,2^. IE epidemiology has changed over the past decades. Increased employment of cardiovascular surgery, prosthetic valves, cardiac implantable electronic devices (CIEDs), and long-term intravascular access has led to a growing proportion of healthcare-associated IE, reported in 25%–30% of cohorts^3,4^. Older adults are increasingly affected, with specific features and poorer outcomes compared with younger populations^2^.

Microbiological patterns also vary geographically. *Staphylococcus aureus* is now the leading cause in most series, followed by *S. viridans* group, coagulase-negative *Staphylococcus*, and *Enterococcus* spp.^2,5^. Blood culture–negative IE remains a diagnostic challenge, accounting for up to 31% of cases^2,6,7^.

While large registries such as the International Collaboration on Endocarditis and the EURO-ENDO study have clarified the global burden, data from middle-income countries remain scarce. In Brazil, recent studies report overall mortality around 22.3%^8,9^. According to national health system data (DATASUS), 19,947 IE-related deaths were registered from 2012 to 2022^9^. However, most available reports are single-center, with heterogeneous populations and limited assessment of prognostic factors. Given this scenario, this study describes the clinical and microbiological characteristics of patients with suspected infective endocarditis admitted to a Brazilian tertiary referral center and identifies independent predictors of in-hospital mortality in this cohort.

## MATERIALS AND METHODS

A descriptive, observational, retrospective cohort study was conducted at Mario Covas Hospital, a public tertiary university hospital affiliated with Centro Universitario FMABC in Santo Andre city, Sao Paulo State, Brazil. The hospital is a referral center for cardiology and cardiovascular surgery with 313 beds, including intensive care units, and performs over 500 cardiac surgeries annually.

The study included all adult patients (≥18 years) admitted between January 2012 and December 2022 with suspected IE. Cases were identified via hospital electronic medical records using ICD-10 codes I33.0, I33.9, I38, and I39.8, and by reviewing all patients who underwent valve replacement surgery with mechanical or biological prostheses during the study period. Patients referred from other hospitals with a prior confirmed IE diagnosis were also eligible. Exclusion criteria included age below 18 years, valve replacement without suspicion of IE, and absence of electronic medical records.

Comorbidities were defined according to standard criteria: heart failure (HF) was classified independently of ejection fraction, following the Brazilian HF guidelines; coronary artery disease (CAD) was confirmed by electrocardiographic or imaging evidence of ischemia or by prior cardiology diagnosis; hypertension (HTN), diabetes mellitus (DM), chronic kidney disease (CKD, dialytic or non-dialytic), rheumatic heart disease, and congenital heart disease (CHD) were classified according to specialty society guidelines^2,10-15^. Additional risk factors included prior IE, prosthetic valve use, pacemaker or implantable cardioverter-defibrillator, and people who inject drugs (PWID). Complications like heart failure, septic shock, acute renal failure, conduction abnormalities, periannular abscesses, pseudoaneurysms, and fistulas were recorded when documented in accordance with ESC and EACTS recommendations^2,10^.

Patients were allocated into two groups according to the modified Duke criteria^11^: those fulfilling definite or possible IE criteria composed the IE group, and those in whom the diagnosis was ruled out formed the non-IE group. Clinical definitions followed national and international guidelines^2,10^. Fever was considered when body temperature was >38 °C and documented in medical records, unless attributable to another identified cause. Vascular and immunologic phenomena, including conjunctival hemorrhage, Janeway lesions, Osler nodes, Roth spots, and embolic events in the spleen, lungs, kidneys, or central nervous system, were considered valid if confirmed by specialist evaluation or imaging. The precise timing of embolic events could not be determined due to the retrospective nature of data collection and the inclusion of patients referred from other centers. Glomerulonephritis was defined by clinical assessment and compatible laboratory findings, including hematuria, proteinuria ≥3.0 g/24h, urinary casts, and/or hypertension or edema, and classified according to the Brazilian Society of Nephrology criteria for acute kidney injury and chronic kidney disease staging.

Microbiological evaluation included blood cultures, which were considered valid if collected from peripheral venipuncture (not central lines) within seven days of admission or symptom onset, according to Duke criteria^2,11^. Blood cultures were processed using an automated BACT/ALERT^®^ system (bioMérieux, Marcy-l’Étoile, France), as routinely employed by the institutional AFIP/CEAC laboratory network. Episodes with negative blood cultures were defined as blood culture–negative IE (BCNIE). Surgical specimens (native or prosthetic valves) underwent conventional culture and histopathology. Histological findings were classified following Lepidi *et al*.^16^, and special stains were applied according to Houpikian *et al.* ^17^

Echocardiographic data were obtained from official reports of transthoracic (TTE) and transesophageal (TEE) examinations, documenting vegetations, periannular abscesses, new prosthetic dehiscence, and new significant regurgitation. Cardiac CT and PET/CT were not performed in this cohort during the study period.

Laboratory results were retrieved from the institutional web platform (SES-SP/AFIP, CEAC Zona Norte) and collected at admission or, for inpatients, at symptom onset. The variables analyzed included hemoglobin (≤12 g/dL), hematocrit (≥46%), platelet count (≤150 ×10^3^/mm^3^), leukocyte count (≥12 ×10m^3^/mmm^3^), total bilirubin (≥1.2 mg/dL), arterial lactate (≥1.6 mmol/L), creatinine (≥1.3 mg/dL), C-reactive protein (CRP), and erythrocyte sedimentation rate (ESR/VHS) above the laboratory's upper reference limit, and complement fractions (C3, C4, CH50) or rheumatoid factor (RF) when available.

Primary outcome was in-hospital mortality. Secondary outcomes included surgical indication for valve replacement and occurrence of major complications.

For statistical analysis, categorical variables were presented as absolute and relative frequencies. Associations between categorical variables were tested with Pearson's χ^2^ test or Fisher's exact test; when global differences were detected, standardized adjusted residuals were examined. Binary logistic regression (enter method) was applied to identify independent predictors of in-hospital mortality, with results expressed as odds ratios (OR) and 95% confidence intervals (CI). A two-sided p value <0.05 was considered statistically significant. Analyses were performed on IBM SPSS Statistics, version 20.0 (IBM Corp., Armonk, NY, USA). Variables included in the regression model were selected according to investigator-defined clinical relevance, considering sample size limitations.

### Ethics

This study was approved by the Institutional Review Board of Centro Universitario FMABC (CAAE Nº 69453623.2.0000.0082). Authorization was also obtained from the hospital technical board for electronic record review. Given the retrospective nature of the study, the requirement for informed consent was waived.

## RESULTS

**Figure 1:**
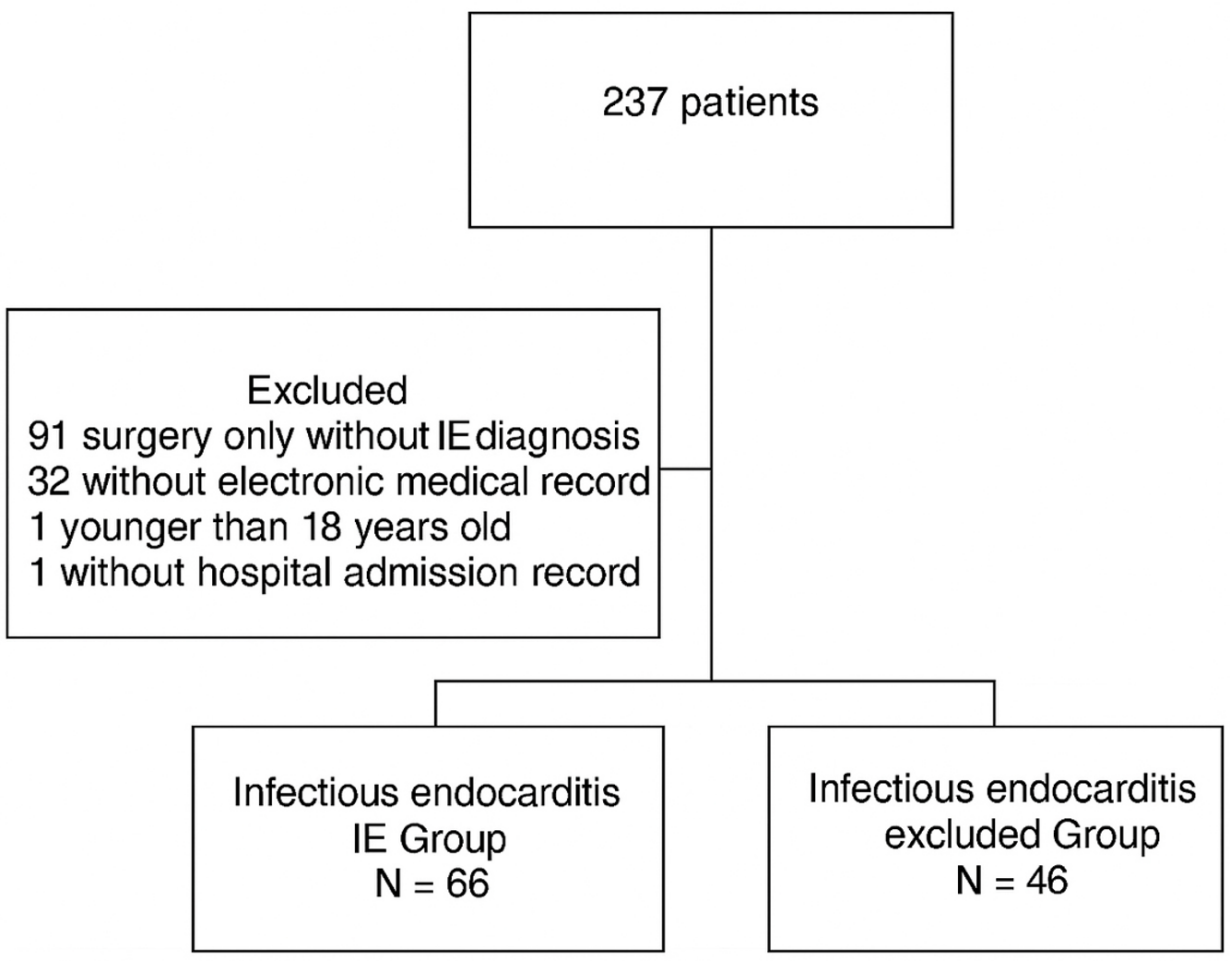
Flowchart of patient evaluation and inclusion in the study.

From January 2012 to December 2022, the center screened 237 patients for suspected IE. Of these, 91 were excluded because they underwent isolated valve replacement surgery without clinical suspicion of IE, 32 due to unavailable electronic records, one for age <18 years, and one for lack of hospitalization data. A total of 112 patients remained eligible, of whom 66 met Duke criteria for definite or possible IE (IE group) and 46 were classified as non-IE (Figure 1).

In the IE group (n=66), mean age was 55.5 years, with 23 patients (34.8%) aged ≥60 years, and 39 (59.1%) were male. Structural heart disease represented the most frequent underlying cardiac condition, observed in 58 of 66 patients (87.8%), including preexisting native valvular disease (48; 75%), prosthetic valve use (11; 16.7%), and congenital heart disease (8; 12.1%). Mitral involvement predominated among native valvulopathies (32; 66.6%), followed by aortic (21; 43%) and tricuspid (14; 29%) lesions.

Most common comorbidities and risk factors included HTN (33; 50%), CAD (15; 22.7%), heart failure (13; 19.7%), hypertrophic obstructive cardiomyopathy (26; 33.3%), CHD (8; 14.8%), previous valve surgery (12; 18.2%), intravenous drug use (6; 9.1%), prior IE (3; 4.5%), and permanent pacemaker implantation (3; 4.5%). During hospitalization, fever (61; 92.4%), vascular phenomena (26; 39.4%), and immunologic phenomena (10; 15.2%) constituted the most common clinical manifestations. Table 1 presents the general baseline clinical and laboratory characteristics of the 112 patients, stratified by group.

**Table 1:** Comparison of the general baseline clinical and laboratory characteristics of the 112 patients, divided by groups, HEMC 2013-2022

Clinical history		Total	IE excluded (N=46)		IE confirmed (N=66)		RP	p value	95%CI
			N	%	N	%			
**Age**							0.41	*0.01* *	0.19-0.89
	< 60 years old	63	20	43.5	43	65.2			
	≥ 60 years old	49	26	56.5	23	34.8			
**Gender**							0.76	*0.29* *	0.35-1.61
	Male	63	24	52.2	39	59.1			
	Female	49	22	47.8	27	40.9			
**CHF** a							1.64	*0.25* *	0.57-4.67
	Absent	93	40	87	53	80.3			
	Present	19	6	13	13	19.7			
**SAH** b							0.70	*0.23* *	0.32-1.50
	Absent	52	19	41.3	33	50			
	Present	60	27	58.7	33	50			
**CAD** c							0.46	*0.05* *	0.20-1.04
	Absent	79	28	60.9	51	77.3			
	Present	33	18	39.1	15	22.7			
**Cardiomyopathy** d							0.94	*0.51* *	0.42-2.07
	Absent	74	30	65.2	44	66.7			
	Present	38	16	34.8	22	33.3			
**Stenosis/regurgitation** e							0.22	*0.01* *	0.06-0.80
	Absent	19	3	6.8	16	25			
	Present	89	41	93.2	48	75			
**Prostetic valve**							0.64	*0.24* *	0.24-1.62
	Absent	90	35	76.1	55	83.3			
	Present	22	11	23.9	11	16.7			
**Valvuloplasty**							0.51	*0.10* *	0.20-1.23
	Absent	86	32	69.6	54	81.8			
	Present	26	14	30.4	12	18.2			
**Congenital Heart Disease**							5.39	*0.08* *	0.62-45.28
	Absent	77	31	96.9	46	85.2			
	Present	9	1	3.1	8	14.8			
**DM** f							0.98	*0.56* *	0.41-2.32
	Absent	83	34	73.9	49	74.2			
	Present	29	12	26.1	17	25.8			
**Rheumatic diseases**							0.84	*0.44* *	0.33-2.10
	Absent	84	35	76.1	49	79			
	Present	24	11	23.9	13	21			
**CKD** g							2.13	*0.10* *	0.76-5.95
	Absent	90	40	87	50	75.8			
	Present	22	6	13	16	24.2			
**Previous IE** h							4.91	*0.11* *	0.57-42.28
	Positive history	100	45	97.8	55	90.2			
	Negative history	7	1	2.2	6	9.8			
**PWID** i		0					2.14	*0.45* *	0.21-21.27
	No use	108	45	97.8	63	95.5			
	Use	4	1	2.2	3	4.5			
**Pacemaker**							1.43	*0.45* *	0.34-6.05
	Absent	103	43	93.5	60	90.9			
	Present	9	3	6.5	6	9.1			
**Laboratory results**		**Total**	**N**	**%**	**N**	**%**	**RP**	^p value^	**95%CI**
**Hemoglobin**							2.28	0.03*	1.02-5.07
	>12g/dL	39	21	46.7	18	27.7			
	≤12g/dL	71	24	53.3	47	72.3			
**Hematocrit**							2.45	0.01*	1.12-5.36
	In reference range	44	24	52.2	20	30.8			
	≥ 46%	67	22	47.8	45	69.2			
**Platelets**							0.95	0.55*	0.38-2.36
	>150,000/mm^3^	85	35	77.8	50	76.9			
	≤150,000/mm^3^	25	10	22.2	15	23.1			
**WBC**							2.75	0.01*	1.25-6.08
	<12,000/mm^3^	46	25	56.8	21	32.3			
	≥12,000/mm^3^	63	19	43.2	44	67.7			
**Total bilirrubin**							1.16	0.61*	0.20-6.55
	< 1,2mg/dL	37	7	77.8	30	75			
	≥1,2mg/dL	12	2	2.2	10	25			
**Arterial lactate**							0.65	0.32*	0.21-2.00
	<1,6 mmol/L	25	10	47.6	15	58.1			
	≥1,6 mmol/L	24	11	52.4	13	41.9			
**Creatinine**							2.78	<0.01*	1.24-6.23
	< 1,3mg/dL	64	33	71.7	31	47.7			
	≥1,3mg/dL	47	13	28.3	34	52.3			
**CPR** j							3.81	0.05*	0.98-14.84
	In reference range	10	6	21.4	4	6.7			
	≥ RR^k^	78	22	78.6	56	93.3			
**ESR** l							1.2	0.68*	0.08-16.43
	In reference range	6	1	33.3	5	29.4			
	≥ RR	14	2	66.7	12	70.6			

Pearson's chi-squared test

* Fischer's exact test

a CHF = Congestive heart failure

b SAH = Systemic arterial hypertension

c CAD = Coronary artery disease

d Obstructive hypertrophic cardiomyopathy

e Moderate or severe stenosis or regurgitation from any cause

f DM = *Diabetes Mellitus;*

g DRC = Chronic kidney disease

h EI = Infective endocarditis

i PWID = People who inject drugs

j CPR = C-reactive protein

k RR = reference range

l ESR = Erythrocyte sedimentation rate.

Blood cultures were obtained for 63 of 66 patients (95.4%), of which 36 (57.1%) yielded a causative pathogen, whereas 27 (42.9%) remained blood culture–negative (BCNIE). *S. aureus* emerged as the leading pathogen (16; 25%), followed by *E. faecalis* (7; 11.1%) and coagulase-negative staphylococci (6; 9.5%). Among patients undergoing surgery, valve specimens underwent culture in 18 (52.9%) and histopathology in 14 (41.1%). Valve cultures resulted negative in 15 (83.3%), whereas histopathological analyses most frequently revealed fibrosis, dystrophic calcification, and degenerative changes (Table 2).

**Table 2:** Results of cultures and echocardiographic findings used in diagnosis, HEMC 2013-2022

Blood and valve cultures		N	
*Staphylococcus aureus* a		16	
*Enterococcus faecalis* a		7	
*Streptococcus viridans* a		3	
*Candida sp* a b		6	
*Staphylococcus epidermidis* a b		4	
*Staphylococcus haemolyticus* a		2	
*Staphylococcus hominis ssp.* a		1	
*Pseudomonas aeruginosa* a		1	
*Acinetobacter baumanii* a		1	
*Escherichia* coli^b^		1	
*Serratia marcescens* b		1	
*Burkholderia cepacia group*.^b^		1	
Negative blood cultures		27	
Negative cardiac valve cultures		15	
**Echocardiogram**		**N**	**(%)**
**TTE**		51	80.90
	Vegetation	35	68.60
	Perivalvular abscess	0	0
	New dehiscence in prosthetic valve	2	3.90
	New valvular regurgitation	9	17.60
	No abnormalities	5	9.80
**Main site affected**			
	Mitral valve	25	49.00
	Aortic valve	18	35.30
	Tricuspid valve	10	19.60
	Pulmonary valve	0	0
	Right atrium	1	7.10
	Left atrium	0	0
**TEE**		32	50.70
	Vegetation	23	71.90
	Perivalvular abscess	0	0
	New dehiscence in prosthetic valve	2	6.20
	New valvular regurgitation	2	6.20
**Main site affected**			
	Mitral valve	15	46.90
	Aortic valve	10	31.20
	Tricuspid valve	4	12.50
	Pulmonary valve	0	0
	Right atrium	5	25.30
	Left atrium	3	15.80

a Blood cultures

b Cultures from surgically removed native or prosthetic valves.

Echocardiography was performed in 63 patients (95.4%): transthoracic echocardiography (TTE) in 52 (80.9%), transesophageal echocardiography (TEE) in 32 (36.5%), and both modalities in 20 (31.7%). TTE identified vegetations in 35 (68.6%), and TEE in 23 (71.9%). TTE identified new significant valvular regurgitation in 9 patients (17%). The valves most frequently involved were mitral (25; 49%), aortic (18; 35%), and tricuspid (10; 19.6%). No pulmonary valve involvement was observed. Findings consistent with IE were documented on the first echocardiographic examination in 44 patients (69.8%). No patient underwent cardiac CT or PET/CT during the study period.

Valve replacement surgery was indicated for 34 of 66 patients (51.5%). Mean preoperative interval was 29 days (range, 1–67), and the mean age of surgical patients was 47.6 years, of whom 9 (26.4%) were ≥60 years. In this subgroup, *S. aureus* was isolated in 8 (23.5%), and 4 (11.7%) presented prosthetic valves at baseline. In-hospital mortality among surgical patients was 12 of 34 (35.2%).

Laboratory abnormalities in the IE group included hemoglobin ≤12 g/dL in 47 (72.3%), hematocrit ≥46% in 45 (69.2%), platelet count ≤150 ×10m^3^/mmm^3^ in 15 (23.1%), leukocyte count ≥12 ×10³/mm³ in 44 (66.7%), total bilirubin ≥1.2 mg/dL in 10 (25%), and serum creatinine ≥1.3 mg/dL in 34 (52.3%). Elevated inflammatory markers were also frequent, with C-reactive protein above the reference range in 56 of 60 tested (93.3%) and erythrocyte sedimentation rate elevated in 12 of 17 tested (70.6%). Complement fractions C3 and C4 were measured in 10 patients (15%), with both reduced in 2. CH50 was assessed in 6 (9%), with one result below the lower reference limit. Rheumatoid factor was seldom tested.

Complications occurred in 30 of 66 patients (45.5%), with acute decompensated heart failure (14; 46.6% of those with complications), septic shock (11; 36.6%), and arterial embolism (20; 30.3% of IE group) presenting most frequently. Embolic events occurred concomitantly in different organs, most often the lungs (15; 75%) and central nervous system (6; 30%). Additional complications included splenic abscess (6; 20%), acute kidney injury (5; 16.6%), pulmonary abscess (2; 6%), and complete atrioventricular block requiring permanent pacemaker implantation (4; 6%). In-hospital mortality in the IE group was 24 of 66 (36.3%). Among the deaths, 16 (66.6%) were in patients aged ≥60 years, 12 (50%) occurred in women, and 12 (50%) occurred after cardiac surgery.

When comparing the IE and non-IE groups in bivariate analyses, IE patients were more likely to present with creatinine ≥1.3 mg/dL (risk ratio [RR] 2.7, 95%CI 1.24–6.23; p<0.01), leukocyte count ≥12 ×10³/mm³ (RR 2.7, 95%CI 1.25–6.08; p=0.01), hematocrit >46% (RR 2.4, 95%CI 1.12–5.36; p=0.01), and hemoglobin ≤12 g/dL (RR 2.2, 95%CI 1.02–5.07; p=0.03).

**Figure 2:**
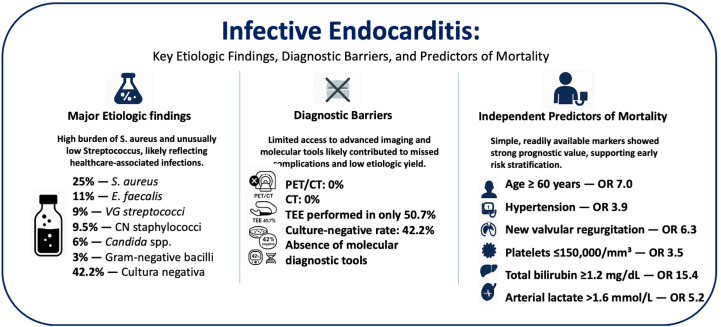
Etiology, diagnostic gaps, and mortality predictors in infective endocarditis.

In the multivariable logistic regression analysis restricted to the IE group, independent predictors of in-hospital mortality included age ≥60 years (odds ratio [OR] 7.0, 95%CI 2.28–21.92; p<0.01), HTN (OR 3.9, 95%CI 1.34–11.60; p=0.01), new valvular regurgitation on echocardiography (OR 6.3, 95%CI 1.15–34.26; p=0.02), platelet count ≤150 ×10³/mm³ (OR 3.5, 95%CI 1.05–11.58; p=0.03), total bilirubin ≥1.2 mg/dL (OR 15.4, 95%CI 1.73–139.65; p=0.03), and arterial lactate >1.6 mmol/L (OR 5.2, 95%CI 1.05–25.96; p=0.04). Table 3 summarizes the variables analyzed and their association with the primary outcome. Figure 2 shows the key etiologic, diagnostic, and prognostic findings.

**Table 3:** Multivariate analysis of variables associated with the primary outcome in EI group patients

		Total	Discharge		Death		Exp (B)	p value	95 %CI
			N	%	N	%			
**Age**							7.08	<0.01	2.28-21.92
	< 60 years old	43	34	81	9	37.5			
	≥ 60 years old	23	8	19	15	62.5			
**SAH**							3.94	0.01	1.34-11.60
	Absent	33	26	61.9	7	29.2			
	Present	33	16	38.1	17	70.8			
**New valvular regurgitation** a							6.3	0.02	1.15-34.26
	Absent	42	27	93.1	15	68.2			
	Present	9	2	6.9	7	31.8			
**Platelets**							3.5	0.03	1.05-11.58
	>150,000/mm3	15	6	14.6	9	37.5			
	≤150,000/mm3	50	35	85.4	15	62.5			
**Total bilirubin**							15.45	<0.01	1.73-139.65
	<1,2 mg/dL	30	19	95	11	55			
	≥1,2mg/dL	10	1	5	9	45			
**Arterial lactate**							5.23	0.04	1.05-25.96
	≤1,6 mmol/L	18	11	78.6	7	41.2			
	>1,6 mmol/L	13	3	21.4	10	58.8			

SAH = Systemic arterial hypertension

a Moderate or severe stenosis or regurgitation from any cause.

## DISCUSSION

This decade-long cohort underscores the persistent complexity of managing IE in resource-limited settings. Predominant structural valvular disease highlights the underlying susceptibility to infection, whereas the central role of *S. aureus* likely reflects a substantial burden of healthcare-associated IE in this setting. The high proportion of blood culture–negative cases reflects enduring diagnostic barriers, whereas the discordance between frequent surgical indication and persistently high mortality illustrates the limits of current therapeutic strategies. Finally, the identification of distinct clinical and laboratory predictors of death emphasizes the need for individualized risk stratification and tailored management approaches.

In this study, 59.1% of IE patients were male, with a mean age of 55.5 years and 39.9% aged ≥60 years, findings consistent with previous reports showing male predominance and similar age distributions in large registries such as GAMES^18^, ICE-PCS^5^, and EURO-ENDO^6^, in which the mean age ranged from 57.9 to 59.2 years^5,6^. International cohorts have described higher proportions of patients aged ≥65 years^5,7,18,19^, whereas a recent national study reported a lower frequency (26.2%)^20^. Age is a major predictor of long-term mortality, with reported rates of 16%–43.2% among those ≥60 years^2,19,20^. In the present cohort, in-hospital mortality was 36.6%, and two-thirds of the deaths occurred in patients aged ≥60 years, consistent with national epidemiology showing that 60.2% of all IE-related deaths in Brazil between 2012 and 2022 occurred in this age group^9^.

Although *S. viridans* has historically been the predominant organism associated with IE in South America^5^, the percentage of *Streptococcus* spp. in the present cohort was unexpectedly low despite the high prevalence of structural heart disease. This pattern likely reflects a predominance of healthcare-associated cases, such as those related to hemodialysis or prolonged hospitalization, which typically favor *S. aureus* in tertiary hospital settings. Globally, *S. aureus* is the most frequently reported pathogen in IE^5,6^, and in this study it accounted for 25% of positive blood cultures, a rate similar to that described in a recent Brazilian cohort (26%)^19^ and slightly higher than that reported in another national study (19%)^21^. Additional organisms identified included *Enterococcus* spp. (11%), *Candida* spp. (6%), coagulase-negative staphylococci (9.5%), *S. viridans* (9%), and gram-negative bacilli (3%), frequencies comparable to international cohorts reporting 3.5% gram-negative bacilli, 2% fungi, and 15.8% *Enterococcus* spp.^6^, with gram-negative bacilli and *Candida* spp. also described in ICU-associated IE^2^. The proportion of culture-negative IE in this study (42.2%) exceeded the 19%–32.3% typically reported in national and international series^5,6,22^, likely influenced by prior antibiotic exposure, although previous antimicrobial use was not assessed and may have led to overestimation.

Structural heart disease was present in 88% of patients, indicating a clear substrate for endocardial infection. The most frequent pre-existing comorbidities among patients with IE were hypertension (50%), CAD (22.7%), HF (19.7%), and CHD (14.8%), values comparable to those reported in EURO-ENDO (HTN 48.3%, CAD 21.5%, HF 23.3%, CHD 11.7%)^6^. That registry also documented the presence of pacemakers in 10.4%, previous IE in 8.8%, non-dialysis CKD in 17.8%, and RRT in 5.2% of patients^6^, findings similar to our cohort for pacemakers (9.1%) and previous IE (9.8%), although CKD (24.2%) and RRT (18.2%) were more common. Moderate to severe valvular stenosis or regurgitation was the predominant predisposing cardiac abnormality (75%), exceeding the 26.3%–34.2% range described in other cohorts^5,6^. Diabetes mellitus has also been associated with increased IE risk. Other studies have shown similar frequencies, including 28% of patients with prosthetic valve IE^23^.

Overall, embolic complications may affect up to 40% of IE patients and are associated with increased morbidity and mortality^10^. In this study, 30.3% of IE patients experienced embolic events during hospitalization, a rate similar to that reported in a prospective multicenter study of 384 IE patients, in which arterial embolism occurred in 34.1%^24^, higher than the 23% observed in the ICE-PCS cohort^5^. Vascular and immunologic phenomena were reported in 39.4% and 15.2% of patients, respectively, findings consistent with previous IE series^2,24^. Classical peripheral stigmata were uncommon, with Osler's nodes (3%), Roth's spots (2%), and Janeway lesions (9%) occurring at frequencies comparable to those described in the literature—1.9%–3%, 1.4%–2%, and 3.5%–5%, respectively^5,6,24^. Glomerulonephritis was identified in 10.6% of patients. Although the 2023 Duke-ISCVID criteria providing a practical definition were not yet available during the study period, retrospective evaluation showed that affected patients fulfilled the updated definition^11^. A lower prevalence of glomerulonephritis (2.9%) was reported in the EURO-ENDO cohort, conducted before introduction of this diagnostic refinement^6^.

Echocardiography was performed in 95.4% of IE patients, predominantly by TTE (80.9%), whereas TEE was used in 50.7%, a frequency similar to reports from developed countries^6^. Cardiac CT and PET/CT were not used, consistent with the limited availability of advanced imaging in South America, where PET/CT use represented only 2.1% of cases in the ICE-PCS study^5^. IE involved native valves in 83.3% of patients, slightly higher than in a Brazilian cohort (77.5%)^19^, and among those with prosthetic valve IE, TEE confirmed typical findings in 60%. Echocardiography identified IE-compatible abnormalities in 69.8% of patients on the first exam, underscoring its core diagnostic role while highlighting the need for complementary imaging when initial findings are inconclusive. Interestingly, the proportion of positive findings appeared relatively similar between TTE TEE in this cohort. This may reflect the predominance of left-sided native valve endocarditis and the presence of vegetations detectable on the initial examination, conditions in which TTE may already provide a high diagnostic yield. Nevertheless, this finding should be interpreted with caution, as the two imaging modalities were not systematically performed in the same patients and the denominators differed between examinations.

Left-sided IE predominated in this cohort (84.4%), mainly affecting the mitral (49%) and aortic (35%) valves, consistent with historical and contemporary reports^19,21^ and with findings from Brazilian studies, including Damasco *et al*.^19^ (mitral 43%, aortic 22%, tricuspid 11%) and another recent cohort reporting even higher rates of mitral (54.5%) and aortic (43.7%) involvement and lower tricuspid prevalence (8.7%)^21^. Vegetations were the most frequent echocardiographic abnormality (68.9%), followed by new significant regurgitation (17.6%), values lower than those observed in larger studies (87.1% and 63.8%, respectively)^5^, but still reflecting the typical spectrum of IE findings.

In our study, 51.5% of IE patients underwent cardiac valve surgery, although the specific surgical indications could not be reliably assessed due to limited documentation in electronic medical records. Mean age of surgical patients was 47.6 years, with a preoperative hospital stay of 29 ± 14.8 days (range 1–67), similar to a national study that reported a stay of 21 ± 19.15 days and a mean age of 50.87 ± 16.15 years^25^. Among those who underwent surgery, 26.4% were aged ≥60 years, men represented 64.7%, and in-hospital mortality reached 35.2%, a multifactorial outcome and higher than the 22.5% reported in national cohorts^25,26^. Mortality rates similar to this range (20%–50%) have been described in studies evaluating only surgically managed IE cases, including an in-hospital mortality of 15.5% reported by Gatti *et al.* ^27^ Most frequent preoperative comorbidities included HTN (50%), DM (20.4%), HF (23.5%), non-dialysis CKD (20.4%), and prosthetic valve use (11.7%), with national data reporting comparable rates for DM (19.3%–26%) and CKD (18%–19.3%) but lower prevalence for HTN (14.8%) despite its association with increased mortality^25,26^. Prosthetic valve use (28%) and HF prevalence (35%) in another Brazilian surgical series^28^ were higher than in this study.

In this study, 36.6% of IE patients died during hospitalization, a rate lower than those reported in other Brazilian series (45.8% and similar values in an additional national cohort)^19,21^, but higher than those observed in large international studies conducted in developed countries, including ICE-PCS (18%), EURO-ENDO (17.1%), and GAMES (28.8%)^5,6,18^. Although IE-related in-hospital mortality remains substantial worldwide, the rate observed here was lower than that described in other developing-country cohorts, yet still markedly above the values typically reported in high-income settings.

Organ dysfunction is defined as an acute increase of ≥2 points in the SOFA score, corresponding to an estimated hospital mortality of 10%^28^. In this study, laboratory abnormalities among IE patients included thrombocytopenia (platelets ≤150,000/mmm^3^) in 23.1%, elevated bilirubin in 25%, and elevated creatinine (≥1.3 mg/dL) in 52.3%. Of these, 52% scored at least 1 SOFA point, and approximately 38% scored ≥2 points, indicating organ dysfunction at admission or at the time IE was suspected. Septic shock, an important IE complication occurring in 5%–10% of cases and considered an indication for urgent surgery, is defined by elevated arterial lactate in conjunction with other criteria^2^. Several biomarkers have been evaluated as diagnostic adjuncts, with CRP and procalcitonin being the most widely studied^2,5,10^. Abnormally elevated ESR may also support the diagnosis, as previously described by Santos *et al*.^29^.

This study presents several limitations. Its relatively small sample size and retrospective design limited subgroup analyses, particularly regarding antibiotic use and surgical indications. Some patients were referred from other facilities, and incomplete medical records may have introduced information bias. Lack of systematic classification of community-acquired versus healthcare-associated endocarditis precluded a more detailed epidemiologic comparison. Moreover, the low prevalence of periannular complications (abscesses, fistulas) in a high-mortality context may indicate underdiagnosis related to limited access to advanced imaging tools, such as transesophageal echocardiography or cardiac CT, reflecting real-world challenges within the public healthcare system. Although several complications were observed during hospitalization, the relatively small number of events limited the ability to reliably assess their independent impact on mortality. Future studies with larger cohorts may better clarify the prognostic significance of these complications in IE patients. Finally, laboratory and imaging data were not uniformly available for all patients, and post-discharge follow-up was not conducted, limiting long-term outcome assessment. Despite these constraints, the study provides valuable insight into the clinical and microbiological features of infective endocarditis in a resource-limited setting.

These findings may have practical implications for clinically assessing infective endocarditis, particularly in resource-limited settings. Readily available parameters such as older age, systemic arterial hypertension, thrombocytopenia, hyperbilirubinemia, and elevated arterial lactate were independently associated with in-hospital mortality and may help clinicians identify patients at higher risk during hospitalization. Additionally, the high proportion of blood culture-negative cases observed in this cohort reinforces the need to strengthen microbiological diagnostic strategies. Future prospective studies with larger cohorts are warranted to validate these predictors and to refine risk stratification approaches in infective endocarditis.

## CONCLUSION

In this decade-long cohort of infective endocarditis from a tertiary referral center in a middle-income country, *S. aureus* emerged as the leading pathogen, blood culture negative cases were frequent, and in-hospital mortality remained high despite surgical interventions. Independent predictors of death included older age, hypertension, new valvular regurgitation, thrombocytopenia, hyperbilirubinemia, and elevated arterial lactate—all of which may serve as clinically useful markers for early risk stratification. These findings underscore the urgent need for improved diagnostic strategies, timely surgical referral, and closer monitoring of high-risk patients in resource-limited settings.

## Data Availability

The anonymized dataset generated during this study is available from the corresponding author upon reasonable request.
